# Identifying Chinese Microblog Users With High Suicide Probability Using Internet-Based Profile and Linguistic Features: Classification Model

**DOI:** 10.2196/mental.4227

**Published:** 2015-05-12

**Authors:** Li Guan, Bibo Hao, Qijin Cheng, Paul SF Yip, Tingshao Zhu

**Affiliations:** ^1^ Key Lab of Behavioral Science of Chinese Academy of Sciences Institute of Psychology Chinese Academy of Sciences Beijing China; ^2^ University of Chinese Academy of Sciences Beijing China; ^3^ HKJC Center for Suicide Research and Prevention The University of Hong Kong Hong Kong SAR China (Hong Kong); ^4^ Key Lab of Intelligent Information Processing of Chinese Academy of Sciences Institute of Computing Technology Chinese Academy of Sciences Beijing China

**Keywords:** suicide probability, microblog, Chinese, classification model

## Abstract

**Background:**

Traditional offline assessment of suicide probability is time consuming and difficult in convincing at-risk individuals to participate. Identifying individuals with high suicide probability through online social media has an advantage in its efficiency and potential to reach out to hidden individuals, yet little research has been focused on this specific field.

**Objective:**

The objective of this study was to apply two classification models, Simple Logistic Regression (SLR) and Random Forest (RF), to examine the feasibility and effectiveness of identifying high suicide possibility microblog users in China through profile and linguistic features extracted from Internet-based data.

**Methods:**

There were nine hundred and nine Chinese microblog users that completed an Internet survey, and those scoring one SD above the mean of the total Suicide Probability Scale (SPS) score, as well as one SD above the mean in each of the four subscale scores in the participant sample were labeled as high-risk individuals, respectively. Profile and linguistic features were fed into two machine learning algorithms (SLR and RF) to train the model that aims to identify high-risk individuals in general suicide probability and in its four dimensions. Models were trained and then tested by 5-fold cross validation; in which both training set and test set were generated under the stratified random sampling rule from the whole sample. There were three classic performance metrics (Precision, Recall, F1 measure) and a specifically defined metric “Screening Efficiency” that were adopted to evaluate model effectiveness.

**Results:**

Classification performance was generally matched between SLR and RF. Given the best performance of the classification models, we were able to retrieve over 70% of the labeled high-risk individuals in overall suicide probability as well as in the four dimensions. Screening Efficiency of most models varied from 1/4 to 1/2. Precision of the models was generally below 30%.

**Conclusions:**

Individuals in China with high suicide probability are recognizable by profile and text-based information from microblogs. Although there is still much space to improve the performance of classification models in the future, this study may shed light on preliminary screening of risky individuals via machine learning algorithms, which can work side-by-side with expert scrutiny to increase efficiency in large-scale-surveillance of suicide probability from online social media.

## Introduction

### Clinical Features of High Suicide Probability

Identifying individuals with suicide probability at an early stage is vital for suicide intervention and prevention. Over the past few decades, people have dedicated themselves to identifying the characteristics of individuals with high suicide probability. Clinicians found high suicide risk in individuals with physical or psychological disease, for example, cancer, Acquired Immune Deficiency Syndrome, and depression [1-3]. There exists a strong connection between high suicide probability and certain personality traits [4,5]; individuals in Asia under specific age groups were reported to be with potential high risk, such as the elderly (especially those in rural areas) and teenagers [6-9]. As for the emotional level, research has shown that hostility, suicide ideation, negative self-evaluation, and depression are the key indicators of suicide. Although many risk factors have been reported to be correlated with suicide probability, it is still difficult to identify suicidal individuals, since suicidal behavior consists of a constellation of complex factors and everyone is unique [10-12]. Moreover, preventive intervention for high suicide probability individuals is often lagged behind, as efforts to track suicidal individuals in populations are hampered by difficulties in data collection and identification of suicide probability [13].

### Research of Internet Suicide Probability Analysis

As the Internet has become a fast growing platform for social interaction in recent years, there are a large number of social network platforms containing suicide related information, which provide a rich source for monitoring suicide probability [14,15]. Researchers have been trying to figure out suicide features and trends from the Internet [13,16-20], and some have managed to locate certain high suicide risk groups by social network analysis [21]. Nevertheless, to the best of our knowledge, little research has been conducted for identifying high suicide probability of individuals using a constellation of Internet features.

### Research Objective

In this study, we examine the feasibility and effectiveness of identifying high suicide probability microblog users automatically based on Internet accessible data. As the dominant microblog service provider in China, Sina Weibo now has 167 million active users, and more than 100 million posts are published daily [22], which provide rich behavior and linguistic information of individuals for any further analysis. As almost all of Weibo users are 35 or younger, this brings us an excellent opportunity to investigate the suicide risk of Weibo youth. We adopt the Suicide Probability Scale in Mandarin to label the suicide probability level of the Weibo users that participated in our Internet survey, and to determine our target group, for example, participants with high risk. We employed two machine learning algorithms, Simple Logistic Regression (SLR) and Random Forest (RF), to train classifiers to predict individual suicide probability via their profile and linguistic features extracted from Sina Weibo, and evaluated the performance of these classifiers on the labeled target group. We expect that the classifier with the best performance can properly identify high-risk individuals through their Weibo data with acceptable accuracy.

## Methods

### Participants and Procedures

Participants were invited to take part in this Internet survey via three approaches on Sina Weibo: (1) recruiting information was published on our laboratory’s official Sina Weibo account with over 5000 followers. Some of the followers took part in the survey voluntarily; (2) a verified celebrity of Sina Weibo, who is a prestigious psychologist in mainland China and has more than 970,000 followers, retweeted our recruiting information and attracted more participants; and (3) another nonofficial Weibo account had been created to send invitation messages randomly on user’s home page. All participants interested in this survey were asked to log on to the Internet survey system by their Sina Weibo account. After they finished reading and signing an informed consent form specifying the objective of the survey and their rights, they were invited to fulfill a survey on demographic information and mental health status, including the Suicide Probability Scale (SPS) in Mandarin. They received a compensation of 30 Renminbi if they completed the whole survey. Contact information of a national suicide prevention hotline was shown on the survey Web page, and the participants were encouraged to seek help if they felt stressful or suicidal. Ethical considerations of the study have been reviewed and granted by the Review Board of the Institute of Psychology, Chinese Academy of Sciences.

### Participant Exclusion Criteria

A participant screening was conducted to assure the quality of this whole process. First, to comply with ethic code, only participants above 18 years of age would be involved. Next, to decrease the possibility that one fulfilled the survey more than once with different microblog accounts, participants’ Internet Protocol (IP) addresses were examined. Survey submissions from the same IP would be eliminated, thus only the first submission would be used. Last, but not least, it was considered that one should have an adequate amount of microblog posts for feature extraction to avoid the “floor effect”, and we only kept participants with more than 100 posts in total.

From May 22th to July 13th, 2014, 1196 Weibo users took part in the survey, 1040 completed the whole survey and 909 of them passed the screening. The final sample pool consisted of 909 Sina Weibo users (561 female, 348 male, mean of age 24.3, SD 5.0).

### Measures

#### Labeling High-Risk Participants

The SPS was developed by Cull and Gill to assess suicide risk of adults and adolescents above the age of 14. Previous studies have verified that SPS could be utilized as an effective screening tool in the community for individual suicide prevention and intervention [23,24]. Liang et al have translated the standardized scale into Mandarin and verified its reliability and validity [25]. SPS consists of 36 self-report questions using a 4-point Likert scale ranging from “none” to “all of the time”. Participants would get a total score of overall suicide probability, as well as scores in four subscales: (1) hostility, (2) suicide ideation, (3) negative self-evaluation, and (4) desperation.

SPS is substantially related to an externally developed index of suicide risk; individuals identified with high suicide probability require further expert scrutiny, or conditional evaluation with family members and friends. The Ontario Hospital Association and Canadian Patient Safety Institute suggested a total raw score of 78 as the cutoff point for high suicide risk [26]. Since there has been no standard norm of SPS score for microblog users in China yet, participants who scored one SD above either the mean of total SPS score or the mean in each subscale score in our Weibo user sample were labeled as high-risk individuals respectively (details in Table 1).

**Table 1:** SPS score distribution and score-based categorization.

Name of scale	Average score	Cutoff for high score class	Cutoff for low score class
	x (SD)	> cutoff point n (%)	< cutoff point n (%)
SPS	69.4 (11.8)	>81 144/909 (15.8)	<58 125/909 (13.8)
Hostility subscale	13.0 (2.5)	>15 137/909 (15.1)	<11 142/909 (15.6)
Suicide ideation subscale	11.5 (3.2)	>14 156/909 (17.2)	<9 94/909 (10.3)
Negative self-evaluation subscale	20.5 (4.4)	>24 173/909 (19.0)	<17 166/909 (18.3)
Desperation subscale	24.6 (4.7)	>29 135/909 (14.9)	<20 110/909 (12.1)

#### Extracting Features From Microblogs

Calling application programming interfaces, provided by Sina Weibo Data Center, allowed all of the publically available digital records of users to be downloaded, from which profile and linguistic features were extracted to train models.

Profile features consist of three types of categories: (1) participant profile or general behavior; (2) user settings; and (3) participant’s microblog behavior.

Category (1) includes: gender; length of username; total number of favorites/followers/follows/friends (mutual follow); length of self-description; length of domain name; count of numbers in domain name; number of openly published microblogs; number of originally published microblogs; number of originally published microblogs with photos; number of originally published posts with URL; numbers of originally published posts with “@”; number of microblogs published between 22:00 and 6:00; number of times that participant used first person plural/singular words; number of total/positive/negative emoticons; and number of days that participant stayed active. To determine positive and negative emoticons, five psychology professionals were recruited to evaluate all 1983 Sina Weibo emoticons. Based on their agreement, 48 positive emoticons and 118 negative emoticons were ultimately identified.

Category (2) includes: whether the user enables private message sending; whether the user allows all users to leave comments; whether the user enables geotagging of their account; and whether the user includes “I” in self-description.

Category (3) includes: the average/maximum/minimum/median number of words in participant’s single microblog; the average number of comments on participant’s single microblog; the average number of times that participant’s single microblog was retweeted; the average number of “likes” for participant’s single microblog; microblog originality (original posts/total posts in public domain); microblog transitivity (posts containing hyperlinks/total posts in public domain); microblog interaction (posts @ other users/total posts in public domain); group reference (the average number of first person plural words per post); self-reference (the average number of first person singular words per post); nocturnal activeness (posts published during 22:00 to 6:00/total posts in public domain); adoption of positive emoticons (the average number of positive emoticons per post); adoption of negative emoticons (the average number of negative emoticons per post); and social activeness (number of friends/number of followers). Ratio data were adopted in many of the Category (3) features to eliminate the impact of time discontinuity, since participants varied in the Weibo active period.

We adopted those features according to three criteria: (1) very few features are raised in previous research. For example, there has been a lot of work focusing on the connection between suicide intention, depressed thinking, and insomnia [27,28], based on this, we adopted the feature of “nocturnal activeness”; (2) some features are defined intuitively, as we think there might exist some kind of relation between the feature and suicide risk (eg, the average number of negative emoticons used per post); and (3) for all the rest, they seem to be common, but important, and we should pay attention to them. Although they have never been mentioned, it is possible that they turn out to be useful for identifying suicide risk.

Using Simplified Chinese Micro-blog Word Count Dictionary (SCMBWC), a Chinese version of Language Inquiry and Word Count [29], which is an effective lexicon for Weibo text analysis [30], linguistic features were extracted. There are 88 features in SCMBWC, covering basic categories in Chinese linguistics such as language process, psychological process, person concern, and oral language. TextMind, a Chinese text analysis system [31], was used in this study to carry out the task of linguistic feature extraction [30].

### Modeling

#### Methodology for Modeling

We built our models on a training set and then evaluated them on a hold-out test set. To do so, we first divided all the participants into three classes. As mentioned above, participants scoring one SD above the mean (mean+1SD) were labeled as high-risk individuals. Accordingly, participants scoring below mean-1SD were labeled as low-risk ones, and those scoring in between were labeled as medium-risk ones. Intuitively, there may exist significant difference in behavioral and linguistic features between high-risk individuals and low-risk ones, thus, models built upon these two groups might capture the appropriate patterns to differentiate high-risk individuals from low-risk ones. To ensure model applicability for the general Weibo user crowd, the proportion of each class in a test set follows the same distribution of the whole participant sample, in which case the performance of models can be genuinely reflected.

Therefore, the training sets are from two extreme groups only, but test sets consist of participants in all three groups, since we want to test the performance of the model in a real world scenario. Here, we run training and testing by 5-fold cross validation. Each training set consisted of 80% of the high-risk and low-risk individuals (suicide probability, 216/269; hostility, 224/279; suicide ideation, 201/250; negative self-evaluation, 272/339; and desperation, 196/245), and each test set consisted of 20% of high-risk, medium-risk, and low-risk individuals (181/909). Both training set and test set were randomly generated 5 times from the whole participant pool to balance the variance of stratified random sampling.

#### Modeling Algorithms and Performance Metrics

There were two machine learning algorithms that were employed for training classification models, SLR and RF. SLR is a type of probabilistic classification model which is a special case of linear model with binary dependent variable. RF is an ensemble method, training multiple decision trees and the final result is the mode of all decision trees’ outputs. The two algorithms have both been used in previous research to triage health problems [32-36]. To evaluate the models, three classic performance metrics were used: (1) Precision (number of true positives/total number of instances predicted to be positive), (2) Recall (number of true positives/total number of positive instances), and (3) F1 measure, which considers the 1:1 tradeoff between precision and recall to give a balanced view [37].

In addition, we also defined “Screening Efficiency” to measure the capacity of workload saved comparing with traditional clinical suicide scrutiny. Screening Efficiency was calculated as, (total number of instances - total number of instances predicted to be positive)/total number of instances. For example, if there were in total 100 individuals, and 40 of them were prescreened by our model as highly risky, then only 40 of them would have to move forward for expert evaluation, thus the workload we might save should be (100-40)/100*100%=60%. Training and testing of models were all conducted via WEKA, a widely adopted machine learning workbench for data mining [38].

## Results

### User Statistics

The majority of users (873/909, 96.0%) were adults below the age of 35, which is consistent with the current age distribution in Sina Weibo. Table 1 summarizes the score distribution and categorization in the whole participant sample pool for total suicide probability and four subscale dimensions. The sample size of each training set (containing 80% of high-score and low-score users) was summarized as follows: 216/269 for SPS total score, 224/279 for hostility score, 201/250 for suicide ideation score, 272/339 for negative self-evaluation score, and 196/245 for desperation score. The sample size of all testing sets was 181/909 (20% of total users under stratified sampling).

### Evaluation

Tables 2-6 show performance of the models on overall suicide probability, as well as four subscale dimensions. SLR and RF were generally matched in performance of classifying potentially risky individuals. For overall suicide probability, the optimal model output was able to achieve a Recall value of 0.82, and Screening Efficiency varied between 0.32-0.46. For hostility dimension, the optimal model output was able to achieve a Recall value of 0.70, and Screening Efficiency varied between 0.42-0.65. For suicide ideation dimension, the optimal model output was able to achieve a Recall value of 0.84, and Screening Efficiency varied between 0.15-0.33. For negative self-evaluation dimension, the optimal model output was able to achieve a Recall value of 0.74, and Screening Efficiency varied between 0.38-0.55. For desperation dimension, apart from two outputs from SLR that tended to identify all individuals as high score, the optimal model output was able to achieve a Recall value of 0.89, and Screening Efficiency varied between 0.21-0.48. Precision values in model outputs varied between 0.1-0.25, and F1 measures varied between 0.17-0.37.

**Table 2:** Model performance for classifying overall suicide probability.

Classifier	Trial number	Performance metrics			
		Precision	Recall	F1 measure	Screening efficiency
SLR	1	0.13	0.50	0.20	0.38
	2	0.14	0.54	0.23	0.42
	3	0.23	0.79	0.35	0.46
	4	0.13	0.50	0.21	0.41
	5	0.19	0.79	0.31	0.36
RF	1	0.13	0.57	0.21	0.32
	2	0.18	0.75	0.29	0.34
	3	0.20	0.82	0.32	0.36
	4	0.16	0.64	0.26	0.38
	5	0.15	0.64	0.24	0.33

**Table 3:** Model performance for classifying hostility.

Classifier	Trial number	Performance metrics			
		Precision	Recall	F1 measure	Screening efficiency
SLR	1	0.12	0.30	0.17	0.62
	2	0.16	0.37	0.22	0.65
	3	0.18	0.52	0.26	0.56
	4	0.16	0.44	0.24	0.60
	5	0.21	0.70	0.33	0.50
RF	1	0.14	0.56	0.22	0.40
	2	0.17	0.67	0.27	0.42
	3	0.14	0.48	0.21	0.47
	4	0.12	0.44	0.18	0.42
	5	0.14	0.52	0.22	0.44

**Table 4:** Model performance for classifying suicide ideation.

Classifier	Trial number	Performance metrics			
		Precision	Recall	F1 measure	Screening efficiency
SLR	1	0.19	0.81	0.31	0.29
	2	0.22	0.84	0.34	0.33
	3	0.19	0.74	0.30	0.33
	4	0.16	0.65	0.26	0.31
	5	0.20	0.81	0.32	0.30
RF	1	0.17	0.84	0.28	0.15
	2	0.17	0.81	0.29	0.20
	3	0.18	0.84	0.29	0.18
	4	0.17	0.77	0.28	0.21
	5	0.17	0.77	0.27	0.20

**Table 5:** Model performance for classifying negative self-evaluation.

Classifier	Trial number	Performance metrics			
		Precision	Recall	F1 measure	Screening efficiency
SLR	1	0.25	0.68	0.37	0.49
	2	0.24	0.59	0.34	0.53
	3	0.20	0.47	0.29	0.55
	4	0.21	0.62	0.32	0.45
	5	0.24	0.74	0.36	0.41
RF	1	0.22	0.71	0.33	0.39
	2	0.23	0.65	0.34	0.47
	3	0.22	0.65	0.33	0.46
	4	0.22	0.74	0.34	0.38
	5	0.20	0.62	0.30	0.41

**Table 6:** Model performance for classifying desperation.

Classifier	Trial number	Performance metrics			
		Precision	Recall	F1 measure	Screening efficiency
SLR	1	0.15	1.00	0.26	0
	2	0.17	0.89	0.29	0.22
	3	0.15	1.00	0.26	0
	4	0.14	0.48	0.21	0.48
	5	0.15	0.63	0.24	0.36
RF	1	0.14	0.67	0.24	0.31
	2	0.13	0.67	0.22	0.26
	3	0.13	0.56	0.21	0.37
	4	0.10	0.44	0.17	0.37
	5	0.15	0.78	0.25	0.21

## Discussion

### Principal Results and Comparison With Prior Work

The key finding of our study is that a high level of suicide probability along the dimension of hostility, suicide ideation, negative self-evaluation, and desperation can be identified with acceptable performance via the profile and text data of microblog users. It is shown that classification performance was generally matched between SLR and RF. Precision varies from 10% to 25%, Recall varies from 30% to 89%, F1 measures vary from 17% to 37%, and the Screening Efficiency varies from 21% to 65%. The performance of the classifiers seems to depend on the randomization of data between the training and testing sets. For example, the Recall on hostility using SLR varies by 40% (0.30-0.70), but only by 7% for suicide ideation using RF (0.77-0.84). It may suggest that the degree of generalizability is different for the four risk factors measured in subscales; for example, future studies may be designed to verify whether suicide ideation has the greatest potential in identifying individual suicide risk among all the emotional factors.

For any risky individual, suicide prevention and intervention is a continuous process, involving a constantly alternating process of suicide risk evaluation and intervention therapy [39]. The traditional process is both time and effort consuming, and because many suicidal individuals in China don’t actively seek help [39], they are often beyond the reach of professional service. Researchers in the suicide prevention and intervention fields have realized the great potential of Web-based intervention; Internet programs have been developed to help people diagnosed as suicidal [40-42]. Our study aims at providing empirical evidence that a suicide risk evaluation process can be conducted through examining online social media content. A computerized algorithm evaluation can work side-by-side with traditional questionnaire methodology to provide reference information for identifying potentially risky individuals and guide them to further intervention.

As the evaluation result shows, among the three classic performance metrics, Recall is generally higher than the other two. This suggests that the models attempt to retrieve as many risky suicidal individuals as possible, even at the cost of partly increasing false alarm. Considering the severity of the suicide act, we do not want to miss any risky individual. Therefore, Recall is our primary concern in this study. However, low Precision and F1 measure indicate that the current model alone can only serve as a preliminary screening tool for suicide probability. Some of the latest research findings also suggest that even though prediction of psychological problems by machine learning algorithms have advanced in accuracy, they still cannot take the place of expert scrutiny [43-47]. To apply our current findings, we can work together with suicide prevention organizations, the computerized program prescreens Weibo users’ suicide risk and then automatically refer high-risk individuals to such organizations. They will further manually examine and provide intervention services according to their professional assessment.

It is thus of our particular interest to explore to what extent preliminary screening of high-risk individuals via machine learning algorithms can reduce the workload in traditional scale assessment for suicide risk. It is shown from our newly defined metric “Screening Efficiency” that, assuming the proposed models serve at their best performance, currently we are just able to save less than half of the traditional workload in general. Although not directly complementary to Recall, a sign of tradeoff has been revealed in many of the experiment trials between the amount of saved workload for further scrutiny, and the proportion of correctly retrieved high-risk individuals. Combining the model evaluation results, we believe there is still much space for advancement in improving the predictive power of models in successive research. Nevertheless, it has been a good start to concentrate on the progressive attempt of feature extraction, modeling design, and classifier selection.

### Limitations

In order to facilitate the usability of our Internet survey system, we allowed participants to complete the survey discontinuously. In other words, if a participant was interrupted and forced to pause the survey partly completed, the progress could be saved for the next access. We did find a few participants with long fulfilling time, and were unable to tell whether they were interrupted, or other reasons that might potentially bias the value of self-report assessment. This concern calls for the optimization of Internet assessment methodology. Some researchers have already been working on developing short, good quality tools to test suicidal behavior on the Internet [48], but more efforts need to be spent to reduce response burden and improve accuracy for Internet self-report evaluation.

It is natural to wonder whether there are some features with the strongest predictive power among all the proposed features. According to the model outputs of our study, the powerful indicators are not consistent among different models; the predictive features in models with the same algorithm would even appear different among different trials. In addition, the predictive features are often uninterpretable. Although one of the advantages of machine learning is to discover hidden relations that do not fit in with the current knowledge system, we admit that currently we have better knowledge concerning the overall predictive power of modeling than the specific predictive power of a single feature. It is of our interest to consolidate feature systems and to strengthen output interpretation.

In this pilot study, we categorized users into three classes, and particularly labeled those who scored mean+1SD as high-risk individuals to indicate that they are more likely in need of careful clinical evaluation of suicide risk. Because there has been no norm group with regard to suicide probability scores among China’s Sina Weibo users, we are aware of the possibility of potential bias with regard to this user sample and the based cutoff points for high suicide probability. For future studies that intend to advance in the suicide Internet research in China, they may investigate the localization of this measuring tool into a specific Internet group.

### Conclusions

Social media is widely used at the present time. Our study indicates that high suicide probability can be evaluated via the publicized profile and text information of microblog users. Although currently our model is unable to reach sufficient accuracy to provide diagnosis, this innovative approach does shed light on the value of monitoring large-scale populations, and enables detecting potentially suicidal individuals for suicide prevention professionals’ further follow-up. Future studies need to focus on increasing the accuracy of classification, and testing the performance on a larger scope of social media users.

## Abbreviations

CAS: Chinese Academy of Sciences
IP: Internet Protocol
RF: Random Forest
SCMBWC: Simplified Chinese Micro-blog Word Count dictionary
SLR: Simple Logistic Regression
SPS: Suicide Probability Scale
